# 母乳中全氟及多氟烷基化合物污染水平及婴儿暴露风险评估

**DOI:** 10.3724/SP.J.1123.2023.09023

**Published:** 2024-02-08

**Authors:** Haichuan CHEN, Wencheng CAO, Xiaofang LIU, Xiao LIU, Qingyun CHENG, Yan ZHOU, Sheng WEN

**Affiliations:** 湖北省疾病预防控制中心应用毒理湖北省重点实验室, 湖北 武汉 430079; Hubei Provincial Key Laboratory for Applied Toxicology, Hubei Center for Disease Control and Prevention, Wuhan 430079, China

**Keywords:** 全氟及多氟烷基化合物, 母乳, 污染水平, 影响因素, 婴儿暴露风险, perfluorinated and polyfluoroalkyl substances (PFASs), breast milk, pollution level, influencing factors, infant exposure risk

## Abstract

为探究母乳中全氟及多氟烷基化合物(PFASs)的污染水平及婴儿暴露风险,本研究依托2018-2021年湖北应城的出生队列,采用同位素稀释-高效液相色谱-串联质谱法测定了324名产妇母乳中23种PFASs的含量,使用多元线性回归分析人口学特征、饮食习惯对产妇母乳PFASs含量的影响,估算婴儿经母乳的PFASs每日摄入量并评估其暴露风险。结果表明,母乳中23种PFASs在0.2~100 ng/mL范围内线性关系良好,相关系数均大于0.992,检出限为5~42 pg/mL,定量限为15~126 pg/mL,回收率为65.6%~108.1%,相对标准偏差为1.6%~12.8%。全氟辛烷磺酸(PFOS)、全氟辛酸(PFOA)和全氟己烷磺酸(PFHxS)为主要污染物,检出率均高于80%,中位数分别为200.7、63.5和25.2 pg/mL,其余化合物检出率均低于45%。多元线性回归结果表明,产妇年龄大、腌制食品摄入频率高可能与母乳PFASs暴露水平增高有关,豆类食品摄入频率高可能与母乳PFASs暴露水平降低有关。婴儿经母乳PFOS、PFOA和PFHxS的每日估计摄入量中位数分别为25.1、7.9和3.2 ng/(kg·d),表明部分婴儿经母乳摄入的PFASs健康风险值得关注。

## Abstract (en)

The purposes of this study are to explore the contamination levels of perfluorinated and polyfluoroalkyl substances (PFASs) in breast milk and assess their exposure risk to infants. Based on data from a birth cohort study conducted in Yingcheng, Hubei Province, from 2018 to 2021, the contents of 23 types of PFASs in the breast milk of 324 pregnant women were determined using isotope dilution-high performance liquid chromatography-tandem mass spectrometry. Multiple linear regression was then performed to analyze the effects of various demographic characteristics and eating habits on the concentration of PFASs in breast milk. The daily PFASs intake of infants through breast milk was estimated, and the exposure risk of infants was also assessed. The results revealed that 23 types of PFASs in breast milk had good linear relationships in the range of 0.2-100 ng/mL, with correlation coefficients greater than 0.992. The limits of detection were 5-42 pg/mL, the limits of quantification were 15-126 pg/mL, the recoveries were 65.6%-108.1%, and the relative standard deviations were 1.6%-12.8%. Perfluorooctane sulfonic acid (PFOS), perfluorooctanoate acid (PFOA), and perfluorohexanesulfonic acid (PFHxS), with median concentrations of 200.7, 63.5, and 25.2 pg/mL, respectively, were the main PFASs found in breast milk. The detection rates of these three contaminants were higher than 80%, whereas the detection rates of other compounds were lower than 45%. The results of multiple linear regression showed that older pregnant women and a higher frequency of pickled food intake may be related to increased PFAS levels in breast milk whereas a higher frequency of legume intake may be related to decreased PFAS levels in breast milk. The median estimated daily intakes (EDIs) of PFOS, PFOA, and PFHxS for infants were 25.1, 7.9, and 3.2 ng/(kg·d), respectively. In summary, this study found notable PFAS levels in breast milk in Yingcheng, Hubei Province. Among these PFASs, PFOS, PFOA, and PFHxS were the main contaminants. Maternal age as well as pickled food and legume intake may affect the PFAS level in breast milk. The health risk of PFAS intake through breast milk to some infants is worthy of attention and further study.

全氟及多氟烷基化合物(perfluoroalkyl and polyfluoroalkyl substances, PFASs)是一类含有至少一个完全氟化碳原子的有机氟化物。由于具有防水和防油的能力,PFASs已被广泛应用于各种消费品和工业中^[1⇓-3]^,如用于食品包装材料、消防泡沫、烹饪锅的表面涂层,还有纺织品、纸张、电子设备的保护涂层,以及用作各种清洁剂中的表面活性剂^[4⇓-6]^。已有大量研究表明,这些化合物对哺乳动物具有多种毒性,如肝毒性、脂质代谢破坏、生殖毒性、发育毒性、内分泌毒性和免疫毒性^[7⇓-9]^。PFASs已被我国纳入《重点管控新污染物清单(2023年版)》。

母乳是婴儿成长最安全、最完整的天然食物,为婴儿提供了几乎所有必要的营养,并可以预防多种疾病。世界卫生组织推荐对6月龄以内的婴儿进行纯母乳喂养^[10]^。PFASs能与母乳中的乳蛋白结合^[11]^,母乳中的PFASs对婴儿发育和健康可能存在潜在不利影响,包括甲状腺功能障碍、免疫抑制和神经毒性等^[12⇓-14]^。第三次全国母乳调查(2017-2020年)^[15]^结果表明我国母乳中PFASs主要污染物是全氟辛酸(perfluorooctanoate acid, PFOA)和全氟辛烷磺酸(perfluorooctane sulfonic acid, PFOS),其含量水平中位数分别为61.2 pg/mL和35 pg/mL。

本研究选取湖北应城地区产妇作为研究对象,采样调查点为化工区,通过测定母乳中PFASs的含量,探讨母亲PFASs的内暴露水平及其影响因素,并评估该地区婴儿哺乳期PFASs的暴露风险。

## 1 实验部分

### 1.1 仪器、试剂与材料

Xevo TQ-S Micro液相色谱-三重四极杆质谱(美国Waters公司); XH-B涡旋混匀器(江苏康健医疗用品有限公司); Multifuge X3R冷冻离心机(美国Thermo公司); QYN100-1水浴氮吹仪(武汉泰仕德科技有限公司); ME204/02电子天平(上海梅特勒-托利多仪器有限公司)。

PFASs标准品和定量内标溶液均由Wellington实验室(加拿大Guelph)提供,纯度均>98%。标准品包括:全氟丁酸(PFBA)、全氟戊酸(PFPeA)、全氟己酸(PFHxA)、全氟庚酸(PFHpA)、全氟辛酸、全氟壬酸(PFNA)、全氟癸酸(PFDA)、全氟十一酸(PFUdA)、全氟十二酸(PFDoA)、全氟十三酸(PFTrDA)、全氟十四酸(PFTeDA)、全氟十六酸(PFHxDA)、全氟十八酸(PFODA)、全氟丁烷磺酸(PFBS)、全氟戊烷磺酸(PFPeS)、全氟己烷磺酸(PFHxS)、全氟庚烷磺酸(PFHpS)、全氟辛烷磺酸、全氟壬烷磺酸(PFNS)、全氟癸烷磺酸(PFDS)、全氟十二烷磺酸(PFDoS)、6∶2氯代多氟烷基醚磺酸(6∶2 CI-PFESA)、8∶2氯代多氟烷基醚磺酸(8∶2 CI-PFESA)。13种全氟化合物同位素混合内标(质量浓度均为2000 ng/mL)包括:^13^C_5_-PFHxA、^13^C_4_-PFBA、^13^C_8_-PFOA、^13^C_9_-PFNA、^13^C_6_-PFDA、^13^C_7_-PFUdA、^13^C_2_-PFDoA、^13^C_3_-PFHxS、^13^C_8_-PFOS、^13^C_5_-PFPeA、^13^C_3_-PFBS、^13^C_4_-PFHpA、^13^C_2_-PFTeDA。

乙腈、氨水、甲酸、乙酸铵(色谱纯,美国Thermo公司);甲醇(色谱纯,美国Sigma公司); WAX固相萃取柱(3 mL/60 mg,美国Water公司);尼龙过滤膜(0.2 μm,天津津腾实验设备有限公司)。

### 1.2 样品前处理

采用刘嘉颖^[16]^报道过的母乳前处理方法,稍作改动。取2 mL母乳样品于15 mL聚丙烯离心管中,加入10 μL质量浓度为5 ng/mL的定量内标溶液和6 mL乙腈,涡旋混合,超声15 min,以4000 r/min离心15 min,收集上清液于第2个离心管中,再在第1个离心管中加入6 mL乙腈,进行第2次提取,合并两次提取液用WAX固相萃取柱净化。WAX柱先用2 mL甲醇、2 mL纯水活化,上样后分别用1 mL 2%(v/v)的甲酸水溶液和1 mL 2%(v/v)的甲酸水溶液/甲醇(1∶1, v/v)淋洗,真空抽干WAX柱后再用2 mL甲醇进行第二次淋洗,最后用2 mL 9%(v/v)的氨水甲醇溶液将目标化合物从WAX柱上洗脱下来,N_2_吹干后用50%(v/v)的甲醇水溶液定容至200 μL,用尼龙过滤膜除去杂质后进样测定。

### 1.3 样品测定与质量控制

采用同位素稀释-高效液相色谱-串联质谱法测定母乳中的23种PFASs,色谱柱为ACQUITY UPLC BEH-C18柱(50 mm×2.1 mm, 1.7 μm),仪器条件具体参数见课题组前期发表文献[17]。

实验过程中所有可能与样品接触的实验用具均在使用前用水清洗3次,甲醇清洗3次。在流动相泵和进样阀之间加两根串联的预柱(BEH-C18, 5 mm×2.1 mm, 1.7 μm),从而将仪器的污染峰和样品峰分开,每次进行仪器分析之前需先用甲醇清洗色谱系统,并在实际样品分析之前进样甲醇或超纯水控制系统污染。每20个样品包含1个过程空白对照:用超纯水作为空白样品来监测前处理过程的本底水平,并在计算实际样品PFASs含量时扣除这部分过程空白。

### 1.4 研究对象

以2018年1月至2021年12月在湖北应城开展的出生队列研究为基础,招募来应城人民医院进行产检的孕早期(<16周)孕妇,并逐一完成问卷调查。研究对象纳入标准如下:(1)当地居住超过1年;(2)单胎活产;(3)孕前无糖尿病、甲状腺异常及肝功能异常。最终有324名产妇纳入本研究。产后由当地社区卫生服务中心工作人员进行入户随访,采集母乳。本研究经过湖北省疾病预防控制中心伦理委员会审核通过(批件号2018-006-02),所有参与者均签署了知情同意书。

### 1.5 问卷调查

问卷调查内容包括母亲基本人口学特征如随访时年龄、身体质量指数(BMI)、受教育水平与家庭平均月收入,母亲孕期膳食食用种类如奶类、蛋类、肉类、海鲜类、水果类、蔬菜类、豆类、腌制类、烧烤类、油炸类,以及膳食食用频率(如:不吃、每天吃、每周吃、每月吃)。

### 1.6 统计分析

使用SPSS 26.0进行统计分析,对检出率大于80%的3种PFASs(PFOA、PFOS和PFHxS)及其含量之和(∑3PFASs)进行统计分析。母乳中PFASs浓度水平低于检出限的数据以LOD/ $\sqrt{2}$ 计算。由于PFASs浓度水平不满足正态分布,将其进行自然对数转换后纳入统计模型进行分析。采用Pearson相关分析连续变量(年龄、BMI)与PFASs浓度的关系,采用独立样本*t*检验和单因素方差分析比较分类变量(教育水平、家庭收入和膳食频率)对PFASs浓度的影响,将*P*<0.1的因素纳入最终多元线性回归模型。为了保证每组间有足够的样本量,每种食物的原始频率组被重新划分为两个分类变量(<6次/周,≥6次/周)或者(<1次/月,≥1次/月)代入模型。计算出每个变量的标准化回归系数(*β*)值及其95%置信区间,检验水准均为双侧,α=0.05。根据中国营养学会制定的《中国居民膳食营养素参考摄入量》推荐,6月龄内婴儿体重代表值为6 kg,每日母乳摄入量为750 mL,以此来对0~6月龄的婴儿进行PFASs母乳暴露的健康风险评估。婴儿通过母乳摄入的PFASs每日估计摄入量(estimated daily intake, EDI)为母乳中PFASs的含量乘以每日母乳摄入量,再除以婴儿的体重。最后与欧洲食品安全局(European Food Safety Authority, EFSA)给出的PFASs健康指导值^[18⇓-20]^进行比较。

## 2 结果与讨论

### 2.1 方法的线性关系、检出限、回收率和精密度

本研究检测了23种PFASs,每种PFASs绘制5点校正曲线,范围为0.2~100 ng/mL。校准曲线在该范围内线性良好,相关系数均大于0.992。向空白样品基质中添加标准溶液,以信噪比(S/N)为3时的添加浓度作为检出限,以S/N为10时的添加浓度作为定量限,检出限为5~ 42 pg/mL,定量限为15~126 pg/mL。向空白样品中分别添加低、中、高3个不同浓度水平的标准溶液(0.2、10、100 ng/mL),每个水平重复测定6次,加标回收率为65.6%~108.1%,相对标准偏差(RSD)为1.6%~12.8%。

### 2.2 母乳中PFASs含量水平

本研究中PFOS、PFOA和PFHxS检出率较高,分别为92.6%、96.3%、83.6%,含量的中位数分别为200.7、63.5、25.2 pg/mL,结果见表1。PFNS、PFDS、PFDoS和8∶2 CI-PFESA均未在母乳中检出。Han等^[15]^报道了2017-2020年中国24个省份3531份母乳的PFASs含量水平,其中湖北省母乳PFASs主要污染物为PFOS,其他省份基本上以PFOA占主导,且湖北省母乳PFOS含量(平均数为181 pg/mL)是调查省份中最高的。本研究中母乳PFASs的主要污染物为PFOS,与Han等^[15]^报道的结果一致,PFOS含量高于Han等^[15]^报道的水平。与前些年报道的其他国家的相关数据相比,本研究中PFOS的中位数含量水平高于韩国(2013年,中位数50 pg/mL)^[21]^、西班牙(2015年,中位数66 pg/mL)^[22]^、美国(2019年,中位数30.4 pg/mL)^[23]^、加拿大(2018-2011年,中位数52.6 pg/mL)^[24]^;本研究中PFOA的中位数含量水平高于美国(2019年,中位数13.9 pg/mL)^[23]^、加拿大(2018-2011年,中位数41.3 pg/mL)^[24]^,低于韩国(2013年,中位数72 pg/mL)^[21]^、西班牙(2015年,中位数152 pg/mL)^[22]^。总体来说,湖北应城母乳中PFOS的污染水平相对较高,这可能是由于调查采样点位于化工区。

**表1:** 母乳中PFASs的检出率和质量浓度

Compound	Abb.	Detection rate/%	Median/ (pg/mL)	Average/ (pg/mL)	Range/ (pg/mL)
Perfluorooctanoate acid	PFOA	96.3	63.5	78.5	<LOD-332.7
Perfluorooctane sulfonic acid	PFOS	92.6	200.7	342.9	<LOD-3895.6
Perfluorohexanesulfonic acid	PFHxS	83.6	25.2	40.9	<LOD-419.7
Perfluoroundecanoic acid	PFUdA	42.9	<LOD	8.8	<LOD-600.0
6∶2 chlorinated polyfluorinated ether sulfonate acid	6∶2 CI-PFESA	42.3	<LOD	7.0	<LOD-58.4
Perfluorobutanesulfonic acid	PFBS	36.1	<LOD	17.3	<LOD-253.1
Perfluorobutyric acid	PFBA	23.8	<LOD	55.0	<LOD-1005.6
Perfluorohexanoic acid	PFHxA	21.9	<LOD	17.1	<LOD-207.0
Perfluorododecanoic acid	PFDoA	18.5	<LOD	5.4	<LOD-78.0
Perfluoropentanoic acid	PFPeA	10.5	<LOD	11.0	<LOD-129.3
Perfluorodecanoic acid	PFDA	9.0	<LOD	<LOD	<LOD-46.0
Perfluoroheptanesulfonic acid	PFHpS	7.4	<LOD	<LOD	<LOD-24.9
Perfluorononanoic acid	PFNA	3.4	<LOD	<LOD	<LOD-43.3
Perfluoro-1-hexadecanoic acid	PFHxDA	3.4	<LOD	9.6	<LOD-361.0
Prefluoroheptanoic acid	PFHpA	1.2	<LOD	<LOD	<LOD-100.0
Perfluorotridecanoic acid	PFTrDA	0.9	<LOD	<LOD	<LOD-158.3
Perfluoropentane sulfonate acid	PFPeS	0.9	<LOD	<LOD	<LOD-52.0
Perfluoro-1-octadecanoic acid	PFODA	0.3	<LOD	<LOD	<LOD-20.7
Perfluorodecanesulfonic acid	PFDS	0.3	<LOD	<LOD	<LOD-35.8

### 2.3 母乳中PFASs含量的影响因素

本研究共纳入324名产妇,产妇平均年龄为(30.2±4.2)岁,受教育水平大多数(77%)在大学以下,家庭月收入大多数(82%)超过3000元,BMI为(22.5±3.1) kg/m^2^。母乳中的PFASs含量与各种因素的多元线性回归结果见表2。产妇年龄与PFOS(*β*=0.043, 95%CI: 0.012~0.0074)、PFHxS(*β*=0.026, 95%CI: 0.001~0.051)和∑3PFASs(*β*=0.029, 95%CI: 0.006~0.052)均呈正相关。李潇等^[25]^也报道了北京市产妇的年龄与母乳中PFOS含量呈正相关。PFASs是一种持久性有机污染物,生物半衰期长达4~5年^[26]^,可随时间在人体内蓄积,而且随着年龄的增大,人体代谢变慢,从而更容易蓄积。豆类摄入频率高的产妇母乳PFOS含量比摄入频率低的产妇低(*β*=-0.444, 95%CI: -0.838~-0.05), Tian等^[27]^也发现豆类食用频率高与孕妇血清PFASs暴露水平低有关。动物源性食品是人体PFASs暴露的重要来源^[28⇓⇓-31]^,而豆类等素食食物的摄入增加可能会竞争性地减少动物源性食品的摄入量,从而降低人体PFASs高暴露的可能性。腌制食品摄入频率高的产妇母乳PFHxS含量比摄入频率低的产妇高(*β*=0.315, 95%CI: 0.024~0.606),目前无相关文献有类似报道,需要进一步研究。其他因素的分析结果均无统计学意义。

**表2:** 母乳中PFASs含量影响因素的多元线性回归结果(*n*=324)

Influencing factor	*β* (95%CI)			
	ln PFOA	ln PFOS	ln PFHxS	ln ∑3PFASs
Age	0.001 (-0.018, 0.020)	0.043 (0.012, 0.074)^**^	0.026 (0.001, 0.051)^*^	0.029 (0.006, 0.052)^*^
Monthly income/￥				
<3000	0	0	0	0
≥3000, <5000	-0.024 (-0.252, 0.204)	-0.280 (-0.648, 0.087)	-0.112 (-0.405, 0.182)	-0.201 (-0.473, 0.071)
≥5000	0.038 (-0.191, 0.268)	-0.350 (-0.720, 0.019)	-0.100 (-0.395, 0.194)	-0.241 (-0.515, 0.032)
Milk				
<6 times/week	0	0	0	0
≥6 times/week	0.024 (-0.215, 0.264)	-0.064 (-0.450, 0.322)	0.178 (-0.130, 0.486)	-0.009 (-0.294, 0.277)
Egg				
<6 times/week	0	0	0	0
≥6 times/week	0.243 (-0.017, 0.504)	0.265 (-0.154, 0.685)	0.235 (-0.099, 0.569)	0.257 (-0.053, 0.568)
Meat				
<6 times/week	0	0	0	0
≥6 times/week	0.052 (-0.221, 0.325)	0.169 (-0.272, 0.609)	0.015 (-0.336, 0.366)	0.106 (-0.220, 0.432)
Fruit				
<6 times/week	0	0	0	0
≥6 times/week	-0.141 (-0.358, 0.075)	-0.023 (-0.371, 0.326)	0.128 (-0.150, 0.406)	-0.005 (-0.263, 0.254)
Vegetable				
<6 times/week	0	0	0	0
≥6 times/week	-0.243 (-0.573, 0.087)	0.424 (-0.108, 0.956)	0.115 (-0.309, 0.539)	0.162 (-0.232, 0.556)
Bean				
<6 times/week	0	0	0	0
≥6 times/week	0.071 (-0.173, 0.316)	-0.444 (-0.838, -0.05)^*^	-0.025 (-0.339, 0.289)	-0.267 (-0.559, 0.025)
Pickled food				
<1 time/month	0	0	0	0
≥1 time/month	0.174 (-0.052, 0.401)	-0.150 (-0.514, 0.215)	0.315 (0.024, 0.606)^*^	0.020 (-0.250, 0.290)
Barbecue food				
<1 time/month	0	0	0	0
≥1 time/month	0.100 (-0.179, 0.379)	-0.276 (-0.726, 0.174)	-0.349 (-0.707, 0.01)	-0.206 (-0.539, 0.127)
Fried food				
<1 time/month	0	0	0	0
≥1 time/month	0.065 (-0.157, 0.288)	0.164 (-0.194, 0.522)	-0.081 (-0.367, 0.204)	0.102 (-0.164, 0.367)

* *P* < 0.05; ** *P* < 0.01; ∑3PFASs=PFOA+PFOS+PFHxS. *β*: regression coefficient; CI: confidence interval.

### 2.4 婴儿暴露风险评估

婴儿经母乳摄入PFASs的EDI如表3所示,PFOS、PFOA、PFNA和PFHxS的EDI中位数分别为25.1、7.9、2.6和3.2 ng/(kg·d)。EFSA在2008年将PFOS和PFOA的每日耐受摄入量(tolerable daily intake, TDI)分别设定为150 ng/(kg·d)和1500 ng/(kg·d),并得到广泛应用,本研究中PFOA和PFOS的EDI低于其推荐值。然而,基于流行病学调查和毒理学研究,政府机构开始收紧限制。例如,EFSA于2018年将PFOA和PFOS的每周耐受摄入量(tolerable weekly intake, TWI)确定为13 ng/(kg·wk)和6 ng/(kg·wk)。此外,在2020年,他们将4种PFASs(PFOA、PFNA、PFHxS和PFOS)总和的TWI确定为8 ng/(kg·wk)。当使用新的TWI对PFOA、PFOS、PFNA和PFHxS评估时,本研究婴儿的PFASs暴露较高,表明可能存在健康危险,因此通过母乳喂养接触的PFASs风险不容忽视。

**表3:** 婴儿通过母乳摄入PFASs的EDI

Compound	Median	Average	Percentile 95th	Range	
PFOS	25.1	42.9	143	2-	487
PFOA	7.9	9.8	23.4	1.2-	41.6
PFNA	2.6	2.6	2.6	2.6-	5.4
PFHxS	3.2	5.1	19.6	0.8-	52.5
∑4PFASs	44.7	60.4	179.1	6.5-	534.3

∑4PFASs=PFOS+PFOA+PFNA+PFHxS.

## 3 结论

本研究中湖北应城母乳存在PFASs暴露,PFOA、PFOS和PFHxS为主要污染物,其中PFOS的含量水平最高。产妇年龄、腌制和豆类食品的摄入可能会影响母乳PFASs的暴露水平,部分婴儿经母乳摄入PFASs的健康风险值得关注。本研究也存在一定局限性。首先,产妇在怀孕期间,饮食结构可能会发生变化,这可能会导致回忆偏倚,也无法反映产妇长期饮食习惯的累积效应。其次,婴儿的暴露风险评估可能被高估或低估,因为不同婴儿的母乳摄入量存在差异,婴儿的体重变化也很快。今后的研究可以从母乳中PFASs对婴儿的生长发育指标的影响进行探讨,以完善婴儿暴露风险评估。
